# Angiofibroma of Soft Tissue: A Clinicopathological Study of Eight Cases With Emphasis on the Diagnostic Utility of Fluorescence *In Situ* Hybridization Detection for *NCOA2* Rearrangement

**DOI:** 10.3389/fonc.2022.900411

**Published:** 2022-06-27

**Authors:** Canming Wang, Yuqian Fan, Jianguo Wei, Qiujie Xu, Guoqing Ru, Ming Zhao

**Affiliations:** ^1^ Department of Pathology, Cancer Hospital of the University of Chinese Academy of Sciences (Zhejiang Cancer Hospital), Institute of Basic Medicine and Cancer, Chinese Academy of Sciences, Hangzhou, China; ^2^ Cancer Center, Department of Pathology, Zhejiang Provincial People’s Hospital, Affiliated People’s Hospital of Hangzhou Medical College, Hangzhou, China; ^3^ Department of Pathology, Shaoxing People’s Hospital, Shaoxing, China; ^4^ Department of Pathology, Tongxiang Second People’s Hospital, Jiaxing, China

**Keywords:** angiofibroma of soft tissue, fluorescence *in situ* hybridization, *NCOA2* rearrangement, differential diagnosis, *AHRR–NCOA2* fusion

## Abstract

**Background:**

Angiofibroma of soft tissue (AFST) is a rare mesenchymal neoplasm of fibroblastic differentiation. Due to its diverse morphology and the lack of specific immunohistochemistry (IHC) markers, AFST could elicit a broad range of differential diagnosis. Several studies have disclosed in AFST recurrent gene fusions involving *NCOA2*, mainly *AHRR–NCOA2* fusion, providing a useful approach to diagnosing this lesion. We report eight additional cases of this rare entity with emphasis on the diagnostic utility of fluorescence *in situ* hybridization (FISH) detection for *NCOA2* rearrangement.

**Methods:**

Clinicopathological data for eight AFSTs were retrieved. IHC was performed, and FISH was used to detect rearrangements involving *NCOA2*, *DDIT3*, and *FUS* loci.

**Results:**

There were five female and three male patients, ranging in age from 29 to 69 years (median: 55 years). The patients presented mostly with a slow-growing mass in the extremities, with or without intermittent pain. All tumors were located in the lower extremities with three (27.5%) involving or adjacent to the knee joints. Tumor size ranged from 1.5 to 3.8 cm (median: 3.0 cm). Morphologically, the tumors consisted of a proliferation of uniform, bland spindle cells set in alternating myxoid and collagenous stroma with a prominent vascular network composed of countless small, branching, thin-walled blood vessels. Foci of “chicken wire”-like capillaries and medium- to large-sized blood vessels with prominent staghorn morphology were evident in two and four cases, respectively. In addition, sheets of small round cells and foci of cystic changes were observed in one each case. Degenerative nuclear atypia was identified in three cases, while mitosis and tumor necrosis were absent. By IHC, the stromal cells were variably positive for epithelial membrane antigen, desmin, and CD68. By FISH analysis, seven out of eight cases (87.5%) showed *NCOA2* rearrangement, and the remaining one had increased gene copy numbers of intact *NCOA2*; rearrangements involving *FUS* (0/4) and *DDIT3* (0/3) were not identified in the cases analyzed. All tumors were surgically removed, and none had recurrence at follow-up from 5 to 73 months.

**Conclusions:**

FISH analysis for *NCOA2* rearrangement represents a practical method for confirming the diagnosis of AFST on the basis of appropriate histomorphological backgrounds.

## Introduction

Angiofibroma of soft tissue (AFST) is a rare benign mesenchymal neoplasm that was first described by Mariño-Enríquez and Fletcher in 2012 (1) and recently included in the 5th edition World Health Organization (WHO) classification of soft tissue and bone tumors in 2020 (2). Histologically, AFST is characterized by proliferation of uniform bland spindle cells in a fibromyxoid stroma with a prominent vascular network, and variable expressions of epithelial membrane antigen (EMA), smooth muscle actin (SMA), CD34, and desmin by immunohistochemistry (IHC) (1–4). The vascular component in AFST often takes on a complex arrangement of small, branching, thin-walled vessels, contributing to its resemblance to a variety of intermediate and malignant soft tissue neoplasms such as solitary fibrous tumor (SFT), low-grade fibromyxoid sarcoma (LGFMS), myxoid liposarcoma (MLPS), and low-grade myxofibrosarcoma (LGMFS). Cytogenetically, AFST is characterized by the gene rearrangement of *NCOA2*, which results in the formation of a tumor-specific *AHRR–NCOA2* fusion gene in most cases (5–7). In this study, we report eight additional cases of AFST with emphasis on the utility of fluorescence *in situ* hybridization (FISH) analysis of rearrangements involving *NCOA2*, *DDIT3*, and *FUS* for the diagnosis and differential diagnosis of AFST.

## Materials and Methods

### Case Selection

Eight cases of AFST were collected from the files of the Department of Pathology, Zhejiang Provincial People’s Hospital (two cases) and the personal consultation files (six cases) of one of the senior authors (MZ), between January 1, 2016, and December 31, 2021. Clinicopathological and follow-up information was obtained from a review of the electronic medical records and from contacting referring pathologists and clinicians. Hematoxylin and eosin (H&E)-stained slides were reviewed, and the diagnosis was further confirmed according to the criteria defined by the 5th edition WHO classification of soft tissue and bone tumors (2). The study was approved by the institutional review board.

### IHC and FISH Analyses

For IHC analysis, representative sections from formalin-fixed and paraffin-embedded (FFPE) tissue were sliced into 3-µm-thick sections and examined using the Ventana BenchMark XT Autostainer (from Roche, Shanghai, China) according to the manufacturer’s instructions. The following antibodies (all from ZSGB, Beijing, China) were used: epithelial membrane antigen (EMA) (clone UMAB237), STAT6 (clone EP325), CD34 (clone 10C9), S100 protein (clone 15E2E2+4C4.9), SOX10 (clone EP268), CD68 (clone PG-M1), MUC4 (clone 8G7O), NY-ESO-1 (clone E978), smooth muscle actin (SMA) (clone UMAB237), retinoblastoma protein (Rb) (clone OTI3F11), desmin (clone EP15), and Ki67 (clone UMAB107). Appropriate positive and negative controls were run concurrently for all markers. Immunoreactivity was estimated by two observers (MZ and GR). For FISH analysis, commercially available break-apart probes respectively flanking the *NCOA2*, *DDIT3*, and *FUS* loci (all from Anbiping Laboratory, Guangzhou, China) were used on FFPE tissue sections. The FISH assay procedures were performed following the manufacturer’s instructions. One hundred tumor cell nuclei were observed by fluorescence microscopy (ECLIPSE Ni, Nikon, Tokyo, Japan) for quantitative analysis. *NCOA2*, *DDIT3*, and *FUS* break-apart probes were considered to respond positively if more than 10% of tumor cell nuclei exhibited orange-green split signals (defined as the distance between the orange and green signals at least twice the estimated signal diameter). The presence of an isolated single orange signal with an intact orange-green signal (unsplit pair) was also regarded as being positive for gene rearrangement (8). Nuclei were excluded if they had any widespread defects in the nuclear areas, showed a crowded nuclear appearance, or had an obscure nuclear contour. Signals were assessed independently by two of the authors (CW and MZ).

## Results

### Clinical Findings

The clinical findings of the eight tumor cases are summarized in **Table 1**. The patients showed a slightly female predominance (female to male = 1.6:1). The age at presentation was widely distributed and ranged from 29 to 69 years (median: 55 years). The patients presented mostly with a slow-growing mass in the soft tissues of extremities, with intermittent pain documented in two cases. Preoperative duration, known for seven patients, ranged from 2 months to 30 years (median, 5 years). All the tumors were located in the lower extremities (six in the left lateral and two in the right lateral), with three (3/8, 27.5%) involving or adjacent to the knee joints. Pretreatment imaging and intraoperative exploration found that four tumors were subcutaneous, two were subfascial, and two were intramuscular. For the six consultation tumors, the submitted diagnosis by the referring pathologists included AFST (one cases), MLPS (two cases), myxoid SFT (one case), LGFMS (one case), and MFS (one case). All tumors were surgically removed, including simple or marginal excision in five cases and wide resection with clean margins in three cases. None had received additional local or systematic therapy after the surgery. Follow-up information was available for all eight patients with a range of 5 to 73 months (median: 33 months), and no tumor recurrence or metastasis was identified.

**Table 1:** Clinical features and FISH findings of angiofibroma of soft tissue.

Case no.	Sexy/age	Site	Clinical presentations	Size	Follow-up (months)	FISH rearrangements (percentage of split)
1	M/52ys	Left leg	Left leg slow-growing painless subcutaneous mass for 3 months	3.0 cm	NED (73)	Positive: *NCOA2* (25%) Negative: *FUS* (2%)
2	F/29ys	Left leg	Left leg slow-growing painless intramuscle mass for 2 months, with keen joint involvement	3.2 cm	NED (50)	Negative: *NCOA2* (5%, with increased gene copy number of intact *NCOA2* in 11%), *DDIT3* (2%), *FUS* (1%)
3	F/44ys	Left thigh	Left thigh painless mass for 30 years, adjacent to keen joint.	2.0 cm	NED (50)	Positive: *NCOA2* (23%) Negative: *DDIT3* (5%)
4	F/62ys	Left thigh	Left thigh slow-growing mass for 5 year, with pain for 3 days	3.0 cm	NED (38)	Positive: *NCOA2* (30%) Negative: *DDIT3* (4%)
5	F/36ys	Left thigh	Left thigh slow-growing painless subcutaneous mass for 2 year	3.5 cm	NED (28)	Positive: *NCOA2* (17%) Negative: *FUS* (3%)
6	M/69ys	Left leg	Left leg slow-growing mass for 20 years, adjacent to keen joint, with intermittent pain for 1 month	1.5 cm	NED (14)	Positive: *NCOA2* (28%)
7	F/58ys	Right thigh	Right thigh slow-growing painless mass for several months	3.8 cm	NED (6)	Positive: *NCOA2* (20%) Negative: *FUS* (2%)
8	M/68ys	Right leg	Right leg slow-growing painless mass for 10 years	2.5 cm	NED (5)	Positive: *NCOA2* (28%)

F, female; FISH, fluorescence in situ hybridization; M, male; NED, no evidence of disease.

### Pathologic Features

**Figure 1:**
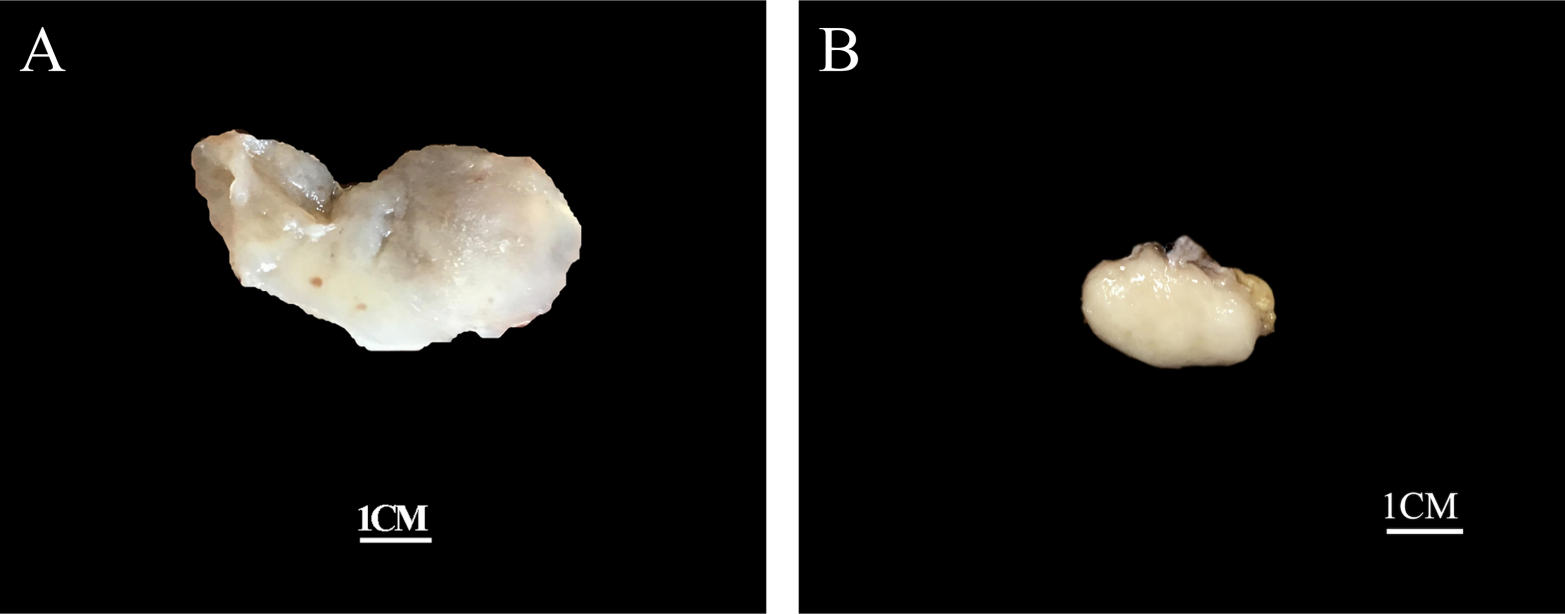
Grossly, the tumors are nodular and well-circumscribed with some showing **(A)** a translucent edematous texture (case 2), **(B)** while others displaying a tan, firm and rubbery cut-surface (case 6).

**Figure 2:**
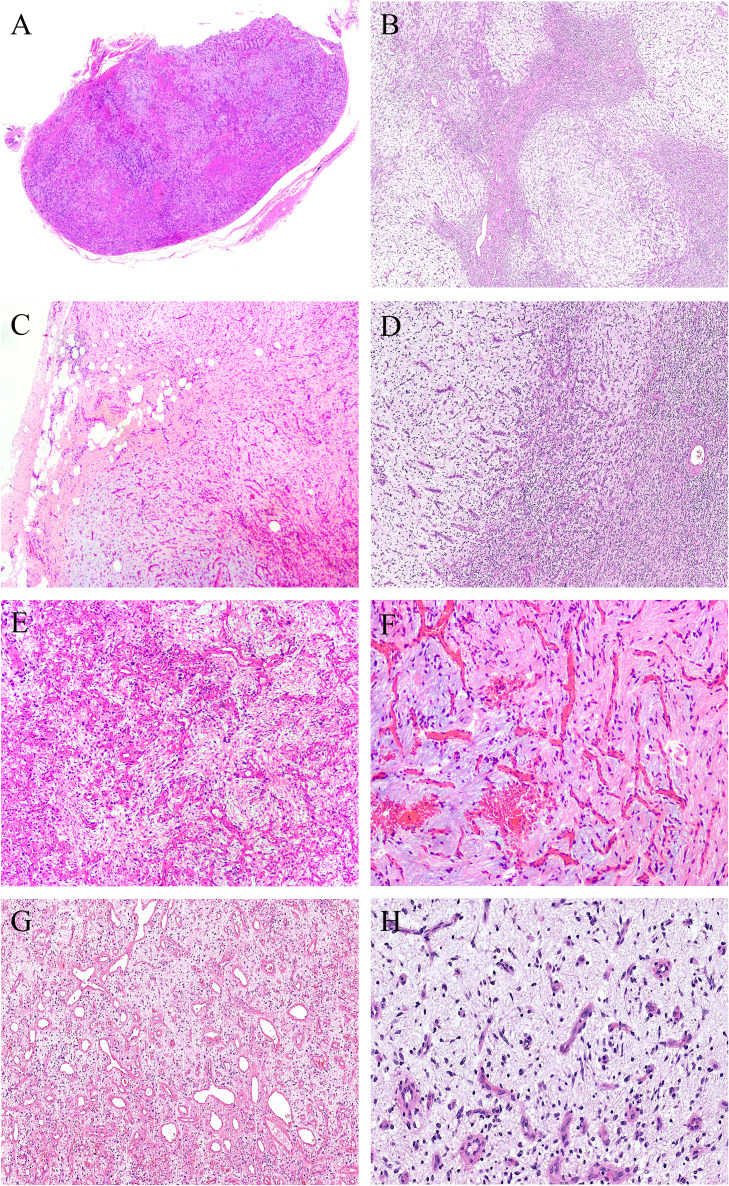
At low power, **(A)** the tumors are well-circumscribed and partially encapsulated (case 6, HE ×15) and **(B)** show a vaguely lobulated pattern of alternating hypocellular, myxoid, and more cellular collagenous areas (case 8, HE ×40). **(C)** Foci of tumor cells infiltration into the surrounding fibroadipose tissues are noted (case 3, HE ×100). **(D)** Prominent vascular network consisting of innumerable, small, thin-walled, frequently slit-like or branching blood vessels (case 8, HE ×120). **(E)** The calibers of the thin-walled blood vessel are sometimes opened and rounded (case 2, HE ×150). **(F)** Foci of plexiform delicate vascular network reminiscent of “chicken wire”-like capillaries characteristic of myxoid liposarcoma are observed; however, the tumor cells are mostly spindle-shaped with bland nuclei, contrasting sharply to the uniform round cells with small lipoblasts in myxoid liposarcoma (case 4, HE ×200). **(G)** Medium- to large-sized blood vessels with prominent staghorn morphology are commonly seen in four cases (case 5, HE ×100). **(H)** The tumor cells have inconspicuous palely eosinophilic cytoplasm and short ovoid or tapering nuclei, with irregular contours, fine chromatin, and indistinct nucleoli (case 8, HE ×200).

**Figure 3:**
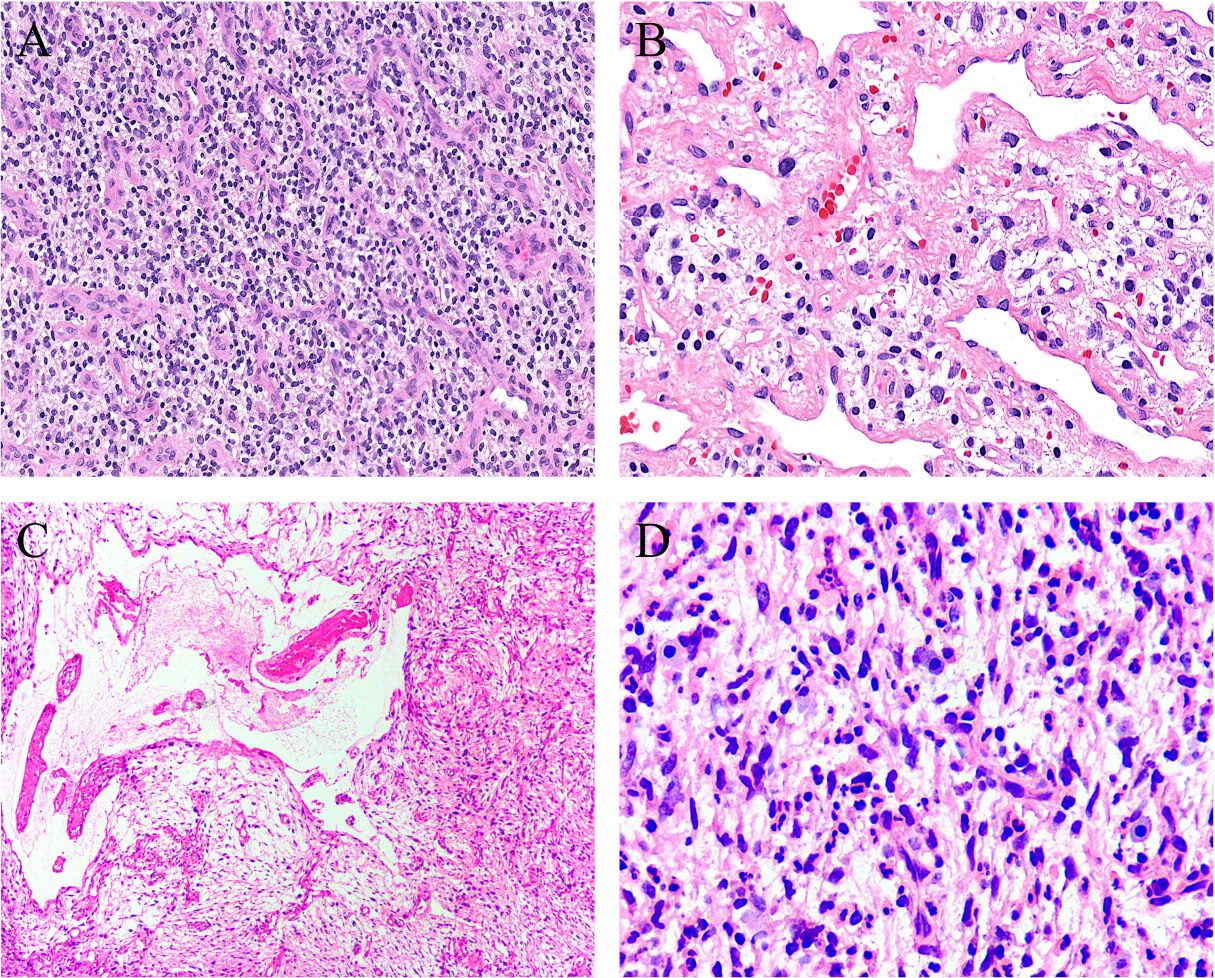
Uncommonly seen features include **(A)** sheets of small round cells separated by a thin-walled vascular network (case 8, HE ×120), **(B)** scattered degenerative nuclear atypia (case 7, HE ×200), **(C)** ischemic cystic change (case 2, HE ×100), and **(D)** foci of prominent neutrophil infiltration (case 3, HE ×200).

Grossly, the tumors were solid nodular and well-demarcated, and the tumor size ranged in greatest dimension from 1.5 to 3.8 cm (median: 3.0 cm). One tumor was grossly multilobulated. The cut surfaces were predominantly firm and rubbery in four cases, whereas in three cases they were edematous and translucent (**Figures 1A, B**). The resection appearances were unknown in the remaining one. Focal hemorrhage and cystic change was grossly noted in one each case. Microscopically, low-power magnification showed that the tumors were well-circumscribed and partially encapsulated and showed a vaguely lobulated pattern of alternating hypocellular, myxoid, and more highly cellular collagenous zones (**Figures 2A, B**). A close examination of the peripheral areas of the neoplasms found minimal infiltration of tumor cells into adjacent soft tissues in three of the eight cases (**Figure 2C** **)**. At medium power, the tumors consisted of a haphazard arrangement of uniform, bland spindle cells with a prominent vascular network composed mainly of countless small, frequently branching, thin-walled blood vessels (**Figure 2D** **)**. The calibers of these blood vessels were usually compressed and slit-like, but sometimes they were opened and rounded (**Figure 2E** **)**. Foci of plexiform delicate vascular networks reminiscent of “chicken wire”-like capillaries characteristic of MLPS were noted in two cases (**Figure 2F** **)**, and medium- to large-sized blood vessels of variably thick walls with prominent staghorn morphology were evident in four cases (**Figure 2G** **)**. At high power, the neoplastic spindle cells have pale eosinophilic cytoplasm and short ovoid or tapering nuclei, often showing irregular contours and fine chromatin with scattered small nucleoli (**Figures 2F, H** **)**. Sheets of small round cells separated by a thin-walled vascular network were noted in one case (**Figure 3A** **)**, and scattered degenerative nuclear atypia with hyperchromasia and nuclear enlargement was identified in three cases (**Figure 3B** **)**, while mitotic counts and tumor necrosis were absent in all cases. Intralesional hemorrhage or ischemic cystic change was noted in three cases (**Figure 3C** **)**. A variably dense inflammatory infiltrate was present in four cases, mainly in the more collagenous areas of the tumors (**Figure 3D** **)**.

### IHC and FISH Results

**Figure 4:**
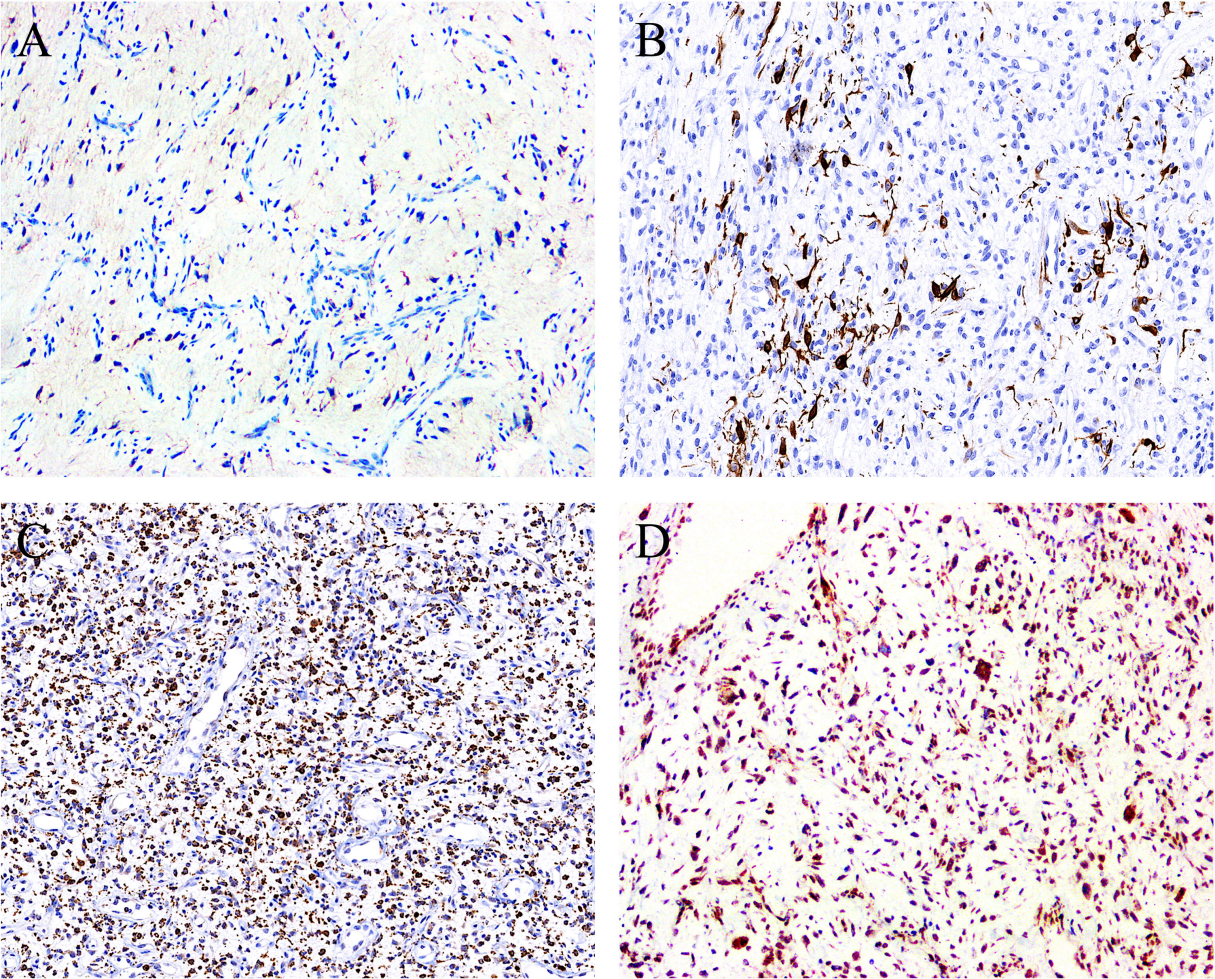
By immunohistochemistry, the stromal cells showed positivity for **(A)** EMA (focal) (case 1, ×100), **(B)** desmin (focal) (case 5, ×120), and **(C)** CD68 (diffuse) (case 7, ×80), with the latter two displaying a dendritic cytoplasmic expression pattern. **(D)** The expression of Rb protein is retained in all cases analyzed (case 2, ×100).

**Figure 5:**
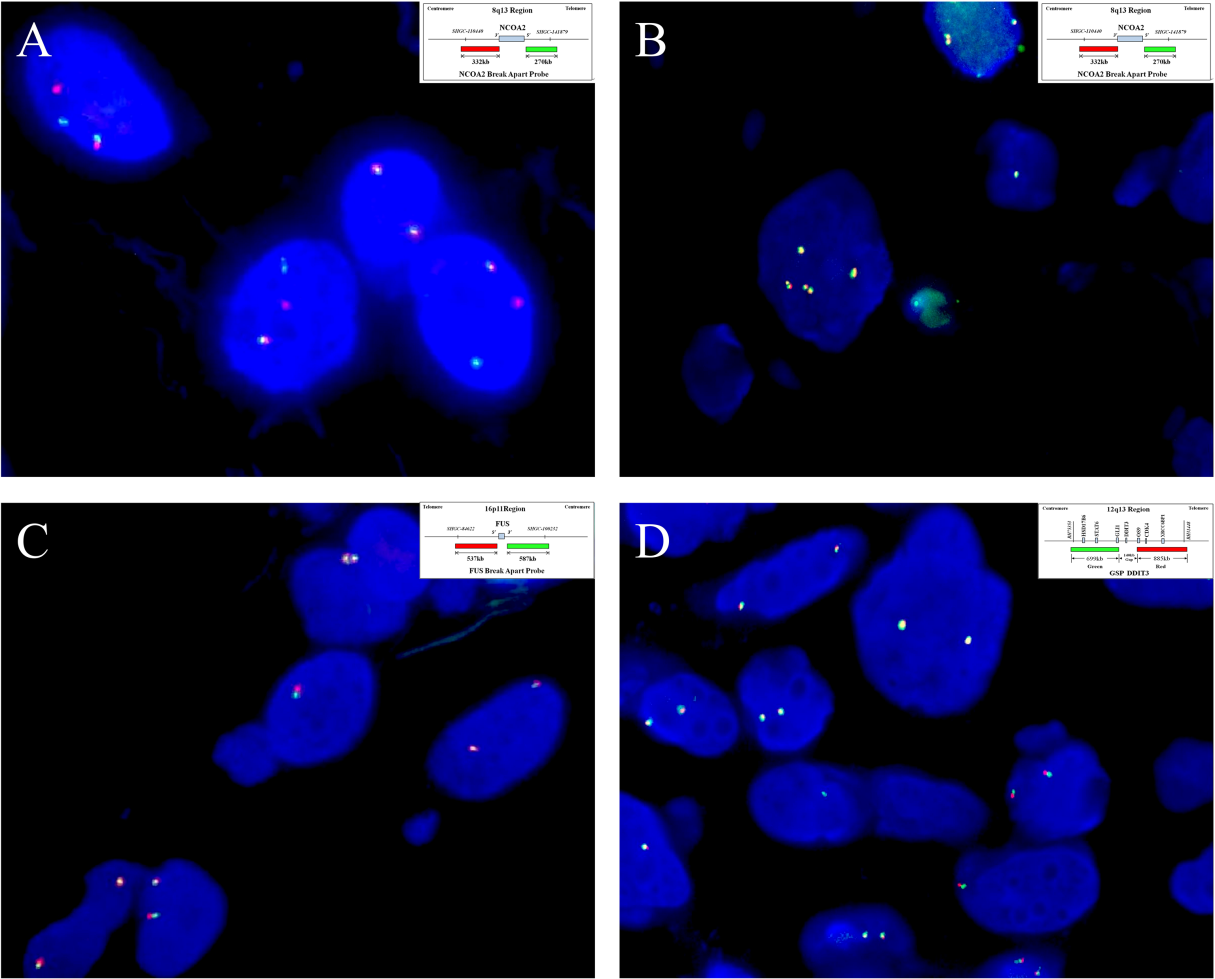
By fluorescence *in situ* hybridization analysis, **(A)** seven of the eight cases analyzed show *NCOA2* gene rearrangement (case 4), **(B)** and the remaining one has increased gene copy numbers of intact *NCOA2* (case 2). (Insets in **A** and **B** indicate schematic diagram of break-apart probes flanking *NOCA2*). Rearrangements involving **(C)** *FUS* (case 7)and **(D)** *DDIT3* (case 3) are not identified in any of the cases examined. (Insets in **C**, **D** indicate a schematic diagram of break-apart probes flanking *FUS* and *DDIT3*, respectively).

On IHC, EMA was focally expressed in three out of eight cases (**Figure 4A** **)**, and desmin was also focally expressed in four out of eight cases, typically showing a dendritic cytoplasmic staining pattern (**Figure 4B** **)**. The expression of CD68 in the stromal cells was present in all eight cases, which was diffuse in five and focal in three cases (**Figure 4C** **)**. Rb was retained in all of the five cases evaluated, exhibiting strong and diffuse reactivity (**Figure 4D** **)**. None of the tumors was positive for nuclear STAT6, while one case displayed a diffuse, non-specific cytoplasmic expression of STAT6. All tumors were completely negative for MUC4, S100 protein, SOX10, and NY-ESO-1. Both SMA and CD34 highlighted the complex vascular network, but they were not expressed on the tumor cells. Ki67 reactivity was very low in most of the cases (<1%), typically with 0 to 3 positive cells/high-power field. By FISH analysis **(** **Table 1** **)**, *NCOA2* rearrangement was positive in seven of the eight cases (7/8, 87.5%) (**Figure 5A** **)**, and the mean percentage of *NCOA2* split was 24% (range: 17% to 30%); the one negative for *NCOA2* rearrangement (percentage of *NCOA2* split: 2%) showed increased gene copy numbers of intact *NCOA2* in 11% of the tumor cells (one to four additional fused signals per nucleus) (**Figure 5B** **)**. Rearrangements involving *FUS* (0/4) (**Figure 5C** **)** and *DDIT3* (0/3) (**Figure 5D** **)** were not identified in any of the cases analyzed.

## Discussion

AFST is a rare soft tissue tumor that was first described by Mariño-Enríquez and Fletcher (1) in 2012 based on a case series of 37 patients. From then on, a total of approximately 80 AFSTs have been reported in the English-language literature to date (1, 5–7, 9–20). In this study, we investigate a series of additional eight AFSTs for their clinicopathologic and molecular genetic features. Our results confirm the female predominance, wide age distribution, predilection for lower extremities as the most commonly affected locations, and occurrence at variable depths of soft tissue levels but often in relation to joints or fibrotendinous structures, as well as their benign/indolent biologic behavior after resection (1, 6, 7). The clinical manifestations of AFST are rather non-specific mostly presenting as a slowly enlarging painless mass of variable duration, although pain and functional limitation have been reported in a few cases (1, 16). The reported cases of AFST ranged in size from 1.2 to up to 12.0 cm (mean: 3.5 cm) in the greatest dimension (4). The diameter in our cohort ranged from 1.5 to 3.8 cm (median: 3.0 cm), falling into the spectrum previously documented.

The histologic features of AFST are very distinctive, with the two most noticeable features being (1) the nodular or vaguely lobular pattern with alternating hypocellular, edematous, or myxoid and more cellular collagenous areas and (2) the complex vascular network composed mainly of countless small, frequently branching, thin-walled blood vessels which tend to be evenly distributed throughout the lesion (1–4). Medium- or large-sized blood vessels with variably thick walls can also be sparsely or rarely diffusely present, frequently with ectatic lumina and a staghorn morphology (1–4). Even in the smallest blood vessels, the lined endothelial cells can easily be appreciable (1). Foci of plexiform vasculature of “chicken wire”-like capillaries closely resembling those seen in MLPS were present in two cases in our cohort, which has rarely been emphasized in the previous reports (1). The neoplastic cells are spindle-, to oval-, or to stellate-shaped with bland nuclei and are haphazardly distributed between the abundant vessels. Degenerative nuclear atypia, often associated with intralesional hemorrhage and/or stromal edema, may be found in a small subset of cases (1, 6, 7, 10, 13, 15), but the mitotic activity does not usually exceed 1/10 high-power fields and the tumor necrosis should be absent (1). Recently, Yamada et al. (6) have described several uncommonly seen histologic features in 13 cases of AFST including depositions of osteoid-like collagen, aggregates of foamy histiocytes, prominent fibrous stroma, nodule in nodule structure, cystic change, and extravasation of red blood cells, further broadening the histomorphology spectrum of this rare entity. Additional morphological findings that have occasionally been reported include hypercellular storiform growth pattern (12) and the presence of multinucleated tumor cells (10, 13). Immunohistochemically, AFST exhibits a non-specific and fibroblastic profile. According to previous studies, approximately one-half of cases showed focal positivity for EMA (1, 3, 5–7). Desmin and SMA have shown focal positivity in 22% and 10% of cases, respectively (1, 3, 5–7, 9). CD34 positivity is rare which has been found in 7% of cases; on the other hand, STAT6, S100 protein, MUC4, and cytokeratins are consistently negative (1, 3, 5–7, 9, 13). Moreover, the expression of CD163 and the estrogen receptor has been observed in 100% of cases in the study by Yamada et al. (6). Our cases showed immunophenotypic features consistent with those described above.

Cytogenetically, AFST is characterized by balanced chromosomal translocation t (5;8) (p15;q13) resulting in formation of the tumor-specific fusion gene *AHRR–NCOA2* (1, 5–7, 9, 14). AHRR is a putative tumor suppressor and regulates the activity of AHR, while NCOA2 functions as a transcriptional coactivator for nuclear hormone receptors. Although the function of the AHRR–NCOA2 chimeric protein still remains to be elucidated, activation of the AHR signaling due to the retained transcriptional activation domain of NCOA2 has been hypothesized to play a vital role in the neoplastic transformation of AFST (5). The *AHRR–NCOA2* fusion has been identified in 60%–80% of AFSTs according to the previous studies (5–7). In addition, alternative fusion events involving *GTF2I–NCOA2*, *NCOA2–ETV4*, *GAB1–ABL1*, and *ETV4–AHRR* have been reported in isolated cases (7, 11, 17). Currently, no link has been established between the genotype and the phenotype. Studies have proven that RT-PCR and direct sequencing are specific methods for detection of the molecular alterations in AFSTs (5–7); however, FISH may be a more sensitive method (6, 13). In accordance with the series investigated by Sugita et al. (6) and Yamada et al. (13), our data suggest that almost all STAFs contain the *NCOA2* rearrangement by FISH analysis, which could serve as a powerful tool for confirming the diagnosis, especially on core needle biopsies when tiny samples are provided. One case without *NCOA2* rearrangement but harboring an increased gene copy number of intact *NCOA2* possessed typical histopathological findings of AFST, suggesting a genetic aberration other than the *NCOA2*-associated fusion genes. It is unclear whether the increase in *NCOA2* gene copy number is another driver event that can contribute to AFST tumorigenesis, but a case of AFST was previously reported by Fukuda et al. (15) in which chromogenic *in situ* hybridization analysis showed *NCOA2* rearrangement accompanied by an increase in gene copy number. However, it should be emphasized that *NCOA2* rearrangement is not unique to SFAT, and *NCOA2* can be the 3′ partner gene in fusions involved in many other bone and soft tissue neoplasms such as mesenchymal chondrosarcoma, alveolar rhabdomyosarcoma, congenital/infantile spindle cell rhabdomyosarcoma, biphenotypic sinonasal sarcoma, and uterine sarcoma with variable sex-cord differentiations (3, 4). Therefore, when using FISH *NCOA2* for the diagnosis of AFST, the appropriate clinicopathological background should be taken into consideration. Although the incidence of *NCOA2* rearrangement by FISH study in AFST is high, the proportion of tumor cells with *NCOA2* split signals may be low. The mean percentage of *NCOA2* split was 24% (range: 17% to 30%) in our cases, in line with the study of 16%–36% by Sugita et al. (6) and 8%–24% by Yamada et al. (13). The reason may be that there are only a few true tumor cells but a relatively large number of non-neoplastic stromal cells, such as dendritic histiocytes which can be highlighted by the immunostains for desmin, CD68, and CD163 (6). This evidence suggests that morphological features need to be taken into account when setting thresholds for the proportion of tumor cells with split signals in FISH analysis of *NCOA2* rearrangement, especially on small biopsy specimens. It should be emphasized that although break-apart FISH has high sensitivity for detecting *NCOA2* rearrangements, it cannot detect the fused partner genes. PR-PCT, Sanger sequencing, or RNA sequencing can be used for further examination of the fusion gene.

Due to the complex vascular structures, haphazardly arranged tumor cells, and the lack of specific IHC markers, the differential diagnostic spectrum of AFST is broad and includes a wide range of soft tissue neoplasms of variable clinical behaviors, such as cellular angiofibroma (CAF), SFT, LGMFS, MLPS, and LGMFS (**Table 2**). The morphological diagnosis of AFST needs to pay attention to its two most distinctive features as mentioned above with the judicious use of specific immunohistochemical markers to exclude its histological mimickers, such as loss of Rb expression/CD34+ in CAF (21), CD34+/STAT6+ in SFT (13, 22), MUC4+ in LGFMS (23), and NY-ESO-1+/S100+ in MLPS (24). Lack of true cytologic atypia, pseudolipoblasts, and brisk mitosis can readily exclude LGMFS. Most notably, the gene rearrangement of *NCOA2* has not been identified in the abovementioned histologically similar neoplasms (13). Therefore, we recommend the detection of *NCOA2* rearrangements by FISH to confirm the diagnosis of AFST in small biopsy specimens or when tumors show variant histological features. In addition to *NCOA2*, we investigated in ASFT the rearrangements of *DDIT3* and *FUS*, which are characteristic of MLPS and LGFMS, respectively (25, 26), and found that no case had rearrangements for these two genes. These data further confirm that molecular genetic testing can be useful for the distinction between ASFT and MLPS and LGFMS, especially on limited tissue samples.

**Table 2:** The differential diagnostic spectrum of angiofibroma of soft tissue.

Tumor type	Important clinical features	Prominent histological features	Positive IHC markers	Diagnostic molecular features	Clinical behavior
AFST	Mostly arising in the subcutaneous soft tissues of extremities, frequently involving or adjacent to large joints	Uniform spindle cells with alternative myxoid and collagenous stroma with complex network of innumerable branching, thin-walled blood vessels	Variable for EMA, CD34, and SMA	*AHRR-NCOA2* fusion (~80%), rarely *GTF2I-NCOA2* or *GAB1-ABL1* fusions	Benign. Rare local recurrence and no metastatic potential
CAF	Typically arising in the superficial soft tissues of the vulvovaginal region	Uniform short-spindle cells in an edematous to fibrous stroma containing short bundles of delicate collagen fibers and numerous small to medium-sized thick-walled blood vessels with rounded, or branching lumina	CD34 (60%), loss of Rb expression of the tumor cells	*13q14* (*RB1*) deletion	Benign. Recurrence or metastasis can be seen for cases with sarcomatous change
SFT	Occurring in both the pleural and extra-pleural locations	Haphazardly arranged of uniform spindle to ovoid cells within a variably collagenous stroma admixed with branching and staghorn-shaped blood vessels that frequently exhibit perivascular hyalinization	STAT6 (nuclear), CD34	*NAB2-STAT6* fusion	Variable. Can be subclassified as low, intermediate, high risk depending on the age, mitosis, tumor size, and necrosis.
LGFMS	Mostly involving in the deep soft tissues of proximal extremities and trunk in young adults, often with a long period of duration	Bland spindle to plump cells with short fascicles or whorling patterns in an alternating hypocellular collagenous and more cellular myxoid stroma with arcades of small vessels	MUC4, EMA (80%), and SMA (30%)	*FUS-CREB3L2* fusion (~90%), less commonly *FUS-CREB3L1* and *EWSR1-CREB3L1* fusions	Low-grade malignant. Can show late recurrence or metastasis
MLPS	Arising in deep soft tissues of the extremities, most often the thigh.	Uniform, round to ovoid cells with variable numbers of small lipoblasts, set in a myxoid stroma with a branching capillary vasculature	NY-ESO-1, and S100 protein (expressed on lipoblasts)	*FUS-DDIT3* fusion (>95%); *EWSR1-DDIT3* fusion (3%)	High-grade malignant. Local recurrence in 25% cases, and distant metastasis in 30%–60% cases.
LGMFS	Mostly arising in the subcutaneous tissues of limbs in elderly patients	Multi-nodular and highly infiltrative growth of hypocellular atypical fibroblastic cells and pseudolipoblasts, in a prominent myxoid stroma with curvi-linear blood vessels.	CD34 and SMA (both are focally expressed)	NA	Low-grade malignant. Local recurrence in 30%–40% cases, with low risk of metastasis

AFST, angiofibroma of soft tissue; CAF, cellular angiofibroma; EMA, epithelial membrane antigen; IHC, immunohistochemistry; LGFMS, low-grade fibromyxoid sarcoma; LGMFS, low-grade myxofibrosarcoma; MLPS, myxoid liposarcoma; NA, not available; SFT, solitary fibrous tumor; SMA, smooth muscle actin.

## Conclusions

AFST is a distinctive benign fibroblastic neoplasm, and simple excision is usually curative. Although it is associated with characteristic features of uniform bland spindle cells within an alternating myxoid and collagenous stroma and a prominent vascular network, there is a morphological overlap with a wide range of soft tissue tumors of variable biologic behavior. FISH analysis for *NCOA2* rearrangement represents a practical method for confirming the diagnosis of AFST on the basis of appropriate histomorphological backgrounds.

## Data Availability Statement

The original contributions presented in the study are included in the article/supplementary material. Further inquiries can be directed to the corresponding author.

## Ethics Statement

The studies involving human participants were reviewed and approved by the Institutional Review Board Committee of Zhejiang Provincial People’s Hospital, Affiliated People’s Hospital of Hangzhou Medical College. Written informed consent for participation was not required for this study in accordance with the institutional requirements.

## Author Contributions

CW, YF, and MZ conceptualized the study concept and design. JW and QX provided the patient samples and clinical data analysis. MZ and GR were in charge of the histologic diagnosis and interpretation of immunohistochemistry. CW performed the molecular experiments and analysis. MZ, CW, and YF drafted the manuscript. All authors contributed to the article and approved the submitted version.

## Funding

This work was supported by the Zhejiang Provincial Natural Science Foundation (LY21H160052) and Zhejiang Provincial Medicine and Health Research Foundation (2019KY020, 2019KY029). The funders did not have any role in the design and conduct of the study, the analysis and interpretation of the data, and preparation of the manuscript.

## Conflict of Interest

The authors declare that the research was conducted in the absence of any commercial or financial relationships that could be construed as a potential conflict of interest.

## Publisher’s Note

All claims expressed in this article are solely those of the authors and do not necessarily represent those of their affiliated organizations, or those of the publisher, the editors and the reviewers. Any product that may be evaluated in this article, or claim that may be made by its manufacturer, is not guaranteed or endorsed by the publisher.
